# The Worth of Words

**Published:** 1904-07

**Authors:** 


### BOOK REVIEWS.

---

THE WORTH OF WORDS. By RALCEY HUSTED BELL, M.D. With an introduction by William Colby Cooper, M.D. Duodecimo, pp. 332. Third edition revised and enlarged. New York: Hinds & Noble. 1904. (Price, $1.25.)

With the assistance of Dr. William Colby Cooper, who writes the introduction, Dr. Bell has sent out a work bearing the above title which will fit into many a hidden nook in the library. The book isn't a big one, but there is a lot of material in it. There is an appendix, too, in which Dr. Bell has a few words to say "as to reviewers." He refers to those who appear to have said unkind things about his work, as "several gentlemanly jaybirds," and "asses." Hoity-toity and forsooth and alackaday! It be wise to closely scan one's review of Dr. Bell's Worth of Words before exposing oneself to the bitterness of his reproach. And yet on second thought why fear reproach when one recalls that, according to Tallyrand, words were made to conceal thought. The object of the work is to call attention to the misuse of words and to point out the most flagrant of common errors. There is much common sense in the book; it might be used to advantage in many newspaper offices and most sewing circles; but Dr. Bell's work in the field of slang is unsatisfying. He does not pretend to give a complete description of all the slang in use, very properly admitting that to attempt to do so would require many volumes; but his brief résumé does not represent the slang of today. Dr. Bell appears to have touched only the bumps on the surface of the subject. He refers to "sky pilot" as an exquisite bit of slang, which it is, and adds that "it is a good word and much better than parson, preacher, minister, dominie, priest." This coming from the author of "The Worth of Words" is almost ghastly. "Sky pilot" is pure slang and is applicable to all denominations, even the itinerant exhorter who has nothing but words and an abiding faith on which to build his hopes. On the other hand "bible banger," another exquisite bit of descriptive slang which Dr. Bell has overlooked, is expressive of those acrobatic ministers of the gospel who literally propound and actually bang the Bible to rags during their discourses. Slang is the dressing of our language; it is characteristic of the people of this country; all nations have slang, and it is characteristic of the American people that their slang fits. It is always apropos. The United States troops probably would have fought as bravely to the air of "Onward Christian Soldiers" in the Santiago campaign, but it is doubtful if there would have been injected into the campaign so much of the ginger of genuine enthusiasm as was shown when the bands played "There'll be a Hot Time in the Old Town Tonight." As noted above, Dr. Bell referred to some of his reviewers as "gentlemanly jaybirds." In his slang department he says: "Jaybird is an epithet of mild ridicule or contempt, according to circumstances," which tells what he thinks of the wicked reviewer. In this connection a slangy one might properly say that Dr. Bell had a "grouch" when he answered his critics. Had he been more severe in his comments on the "gentlemanly jaybird," he would have approached a condition known in slangdom as "red-headed." N. W. W.

